# Comparison of Diffusion-Weighted MRI Reconstruction Methods for Visualization of Cranial Nerves in Posterior Fossa Surgery

**DOI:** 10.3389/fnins.2017.00554

**Published:** 2017-10-09

**Authors:** Brendan Behan, David Q. Chen, Francesco Sammartino, Danielle D. DeSouza, Erika Wharton-Shukster, Mojgan Hodaie

**Affiliations:** ^1^Division of Brain, Imaging and Behaviour – Systems Neuroscience, Krembil Institute, University Health Network, Toronto, ON, Canada; ^2^Division of Neurosurgery, Department of Surgery, Toronto Western Hospital, Toronto, ON, Canada; ^3^Department of Surgery, Institute of Medical Science, University of Toronto, Toronto, ON, Canada; ^4^Department of Neurology and Neurological Sciences, Stanford University, Stanford, CA, United States; ^5^Joint Department of Medical Imaging, University Health Network, Toronto, ON, Canada

**Keywords:** diffusion tensor imaging, extended streamline tractography, constrained spherical deconvolution, fiber orientation distribution, trigeminal nerve, vestibulocochlear nerve

## Abstract

Diffusion-weighted imaging (DWI)-based tractography has gained increasing popularity as a method for detailed visualization of white matter (WM) tracts. Different imaging techniques, and more novel, advanced imaging methods provide significant WM structural detail. While there has been greater focus on improving tract visualization for larger WM pathways, the relative value of each method for cranial nerve reconstruction and how this methodology can assist surgical decision-making is still understudied. Images from 10 patients with posterior fossa tumors (4 male, mean age: 63.5), affecting either the trigeminal nerve (CN V) or the facial/vestibular complex (CN VII/VIII), were employed. Three distinct reconstruction methods [two tensor-based methods: single diffusion tensor tractography (SDT) (3D Slicer), eXtended streamline tractography (XST), and one fiber orientation distribution (FOD)-based method: streamline tractography using constrained spherical deconvolution (CSD)-derived estimates (MRtrix3)], were compared to determine which of these was best suited for use in a neurosurgical setting in terms of processing speed, anatomical accuracy, and accurate depiction of the relationship between the tumor and affected CN. Computation of the tensor map was faster when compared to the implementation of CSD to provide estimates of FOD. Both XST and CSD-based reconstruction methods tended to give more detailed representations of the projections of CN V and CN VII/VIII compared to SDT. These reconstruction methods were able to more accurately delineate the course of CN V and CN VII/VIII, differentiate CN V from the cerebellar peduncle, and delineate compression of CN VII/VIII in situations where SDT could not. However, CSD-based reconstruction methods tended to generate more invalid streamlines. XST offers the best combination of anatomical accuracy and speed of reconstruction of cranial nerves within this patient population. Given the possible anatomical limitations of single tensor models, supplementation with more advanced tensor-based reconstruction methods might be beneficial.

## Introduction

Diffusion-weighted imaging (DWI) is a neuroimaging method that assays the random movement of water molecules to reconstruct the structure of white matter (WM) fibers (Behrens and Johansen-Berg, 2009; Jones et al., 2013; Soares et al., 2013). Within the brain, this movement is affected by structural features such as axons of WM (Mori and van Zijl, 2002; O'Donnell and Westin, 2011). Consequently, the projections of WM tracts can be reconstructed based on their diffusion profiles—a technique termed “fiber tracking” or “tractography” (Conturo et al., 1999; Mori and Barker, 1999; Basser et al., 2000).

Providing detailed information about WM tracts *in vivo* makes tractography an attractive option for neurosurgical practice (Abdullah et al., 2013). Deterministic single diffusion tensor tractography (SDT) has been successfully used to reconstruct representations of large WM fiber tracts within patient populations with supratentorial tumors (Potgieser et al., 2014). While this method tends to provide reliable results when there is one major fiber bundle of interest, it performs less so in areas where multiple WM fiber bundles are present (Wedeen et al., 2008). This is due to its inherent limitation that it assumes each WM voxel has a single fiber orientation (Basser et al., 1994; O'Donnell and Westin, 2011). With an estimated 63–90% of WM voxels containing multiple fiber bundles (Jeurissen et al., 2013), this can be a considerable problem for neurosurgical planning, particularly in terms of following the anatomical course of a nerve when it encounters other fiber bundles.

Given these limitations, attention has turned to developing other methods that satisfactorily deal with the occurrence of multiple fibers in a WM voxel. This can broadly be defined as high angular resolution diffusion imaging (HARDI) which requires more advanced DWI acquisition protocols (Tuch et al., 2002). One option is to glean more information from the original tensor map for fiber reconstruction purposes. EXtended Streamline Tractography (XST) is one such method that is based on two-tensor reconstruction allowing for crossing fiber pathways to be reconstructed (Qazi et al., 2009). XST has been demonstrated to be superior to SDT in its generation of lateral projections of the corticospinal tract, but its efficacy has not been determined in smaller WM tracts.

Another option is to employ methods other than tensor-based approaches to characterize diffusion profiles (Tournier et al., 2011). An emerging popular method involves a non-negativity constrained spherical deconvolution (CSD)-derived estimation of the distribution of fiber bundles per WM voxel—a fiber orientation distribution (FOD) estimate (Tournier et al., 2004, 2007). Similar to XST, this reconstruction method has been demonstrated to illustrate more accurate anatomical depictions of the corticospinal tract (Farquharson et al., 2013) and cerebello-cortical tracts (Palesi et al., 2015), compared to conventional diffusion tensor reconstruction methods. However there has not been as much focus on its application in smaller WM tracts.

SDT has previously been successfully used to image cranial nerves (CNs) in both healthy controls and patients with tumors in the posterior cranial fossa (Kabasawa et al., 2007; Hodaie et al., 2010, 2012; Chen et al., 2011; Gerganov et al., 2011; Roundy et al., 2012; Yoshino et al., 2015; Hilly et al., 2016). Considering that CN projections often pass through regions of multiple fiber populations, and SDT's inability to resolve such arrangements, it is important to evaluate other reconstruction methods that may offer better depictions. Both HARDI-based reconstruction methods, XST and CSD-based streamline tractography, have produced more accurate depictions of larger WM tracts, compared to SDT. As such, we are interested to see whether we would see similar improvements in the visualization of smaller fiber bundles, such as CNs. Here, we aim to compare and contrast the aforementioned three discrete reconstruction methods with regards to the generation of the WM bundles of the trigeminal nerve (CN V), the facial nerve (CN VII), and the vestibulocochlear nerve (CN VIII). CN VII and CN VIII will be hereafter grouped together as one entity—the facial/vestibular complex (CN VII/VIII)—as both fibers tend to be closely aligned with one another in diffusion-weighted scans (Hodaie et al., 2010).

We use a cohort of patients with unilateral posterior fossa tumors to determine which of these aforementioned reconstruction methods provides optimal visualization of the CN fibers. Assessment is made based on the important practical criteria necessary for neurosurgical implementation, including processing speed, anatomical accuracy of fiber representations, and most importantly, the depiction of the relationship between CN fiber bundles and the tumor in the posterior cranial fossa. Furthermore, we use these criteria to determine whether HARDI-based methods, that do not assume one fiber orientation per voxel, show improved visualization parameters compared with single tensor-based models.

## Materials and methods

### Patient demographics

Ten patients (4 male and 6 female, mean age: 63.5, range: 48–85), with cerebellopontine angle tumors, undergoing Gamma Knife radiosurgery treatment, were recruited for this study (for clinical demographic information, see Table 1). Four patients presented with tumors primarily affecting the trigeminal nerve (three meningiomas and one trigeminal schwannoma: P01–P04), with the remaining six presenting with vestibular schwannomas (P05–P10). Institutional Review Board approval was obtained. Patients had not received prior surgical or radiosurgery treatment.

**Table 1:** Patient details with type of posterior cranial fossa tumor and properties.

**Patient**	**Age**	**Tumor type**	**Volume (mm^3^)**	**Maximum dimension (mm)**	**Architecture**
1	54	Petrous Meningioma	2, 201.41	19.1	Solid
2	48	Trigeminal Schwannoma	336.66	12.3	Solid
3	75	Petroclival Meningioma	1, 305.48	18.5	Solid
4	54	Meningioma	983.47	18.9	Solid
5	73	Vestibular Schwannoma	1, 246.07	21.1	Solid
6	71	Vestibular Schwannoma	8, 409.41	28.0	Cystic
7	50	Vestibular Schwannoma	8,020	29.0	Solid
8	71	Vestibular Schwannoma	355.86	11.5	Solid
9	54	Vestibular Schwannoma	1, 266.09	20.0	Solid
10	85	Vestibular Schwannoma	2, 629.72	23.3	Cystic

### Imaging

Magnetic resonance images were acquired using GE Signa HDx 3T scanner with an 8 channel head-coil. MR sequences were acquired from a group of patients with tumors in the posterior cranial fossa. Diffusion weighted images (DWI) were acquired with 1 B0 scan, 60 gradient directions, 3 mm slice thickness and in-plane resolution of 0.9375 × 0.9375 mm, b0 = 1,000 s/mm^2^, TE = 88.6 ms, TR = 17,000 ms, flip angle = 90°, matrix = 128 × 128, number of slices = 44. The scan acquisition time was ~17.5 minutes (min). T1 FSPGR anatomical scans were acquired with 1 mm slice thickness and in-plane resolution of 0.3906 × 0.3906 mm, slice spacing = 1 mm, TE = 3.72 ms, TR = 9.06 ms, flip angle = 12°, matrix = 320 × 320. T1 fast image employing steady state acquisition (FIESTA) scans were acquired with 0.8 mm slice thickness and in-plane resolution of 0.352 × 0.352 mm, slice spacing = 0.4 mm, TE = 2.4 ms, TR = 4.94 ms, flip angle = 37°, matrix = 256 × 256.

### DWI processing

DWI sequences were initially corrected for eddy-current and motion distortions, through an affine transformation, using FLIRT (FMRIB's linear registration tool) in FSL (version 5.0.8) (Jenkinson et al., 2002, 2012). Custom MATLAB scripts were used to correct gradients for motion effects.

### Seed placement

Seeds for CN V were initially placed on retrogasserian portions of the nerve, as this was the most identifiable portion of the nerve and a common target for radiosurgical treatments (Regis et al., 2002; Massager et al., 2004). Seeds for CN VII/VIII were initially placed in the intracanalicular portion, as this was the most identifiable portion, particularly in patients presenting with vestibular schwannomas (P05–P10). Seeds for both CN V and CN VII/VIII were placed bilaterally in all patients. All seeds were mapped so as to incorporate a cross section of the CN of interest and were not more than 20 voxels in size. The same seeding region was used for all three reconstruction methods under consideration.

### Tensor-based methods

#### Single diffusion tensor tractography (SDT)

Images were imported into 3D Slicer version 3.6 (NA-MIC, http://www.slicer.org) (Fedorov et al., 2012) on a Ubuntu 12.04 LTS OS for SDT, where a tensor map was created from the DWI scan using a least-squares method—the “DWI to DTI Estimation” module in 3D Slicer. Tractography parameters were initiated with seed spacing = 0.3 mm, seeding FA threshold = 0.15, stopping FA value = 0.15, curvature threshold = 0.8, minimal length = 5 mm, and integration distance = 0.1. Tractography was performed with the “Labelmap Seeding” module in the 3D Slicer graphical user interface (GUI). Graphical representations of this reconstruction method are detailed elsewhere (Mori et al., 1999).

#### Extended streamline tractography (XST)

Images were analyzed with XST software (Qazi et al., 2009). The tensor map created for SDT was also used for XST. Tractography parameters were initiated with seed spacing = 0.3 mm, stopping FA value = 0.15, stopping linear anisotropy (as measured by C_1_ – Westin et al., 2002) = 0.1, stopping fraction of the chosen tensor component = 0.1, minimal length = 5 mm, and curvature threshold = 0.8. This method was implemented by using the “ten2fiber” command from a modified version of TEEM toolkit (https://github.com/sinkpoint/hodaie-teem). Graphical representations of this reconstruction method are detailed elsewhere (Qazi et al., 2009).

### Fiber orientation distribution(FOD)-based method

#### Constrained spherical deconvolution (CSD)

Images were analyzed with MRtrix3 (Tournier et al., 2004, 2007, 2012). Briefly, the skull of the DWI dataset was removed and a brain mask was formed. A response function, representing the DW signal for a single fiber population, was estimated (using the “dwi2response” function in MRtrix3) and then incorporated into a non-negativity CSD analysis from which a FOD was computed for each WM voxel (using the “dwi2fod” function In MRtrix3). Finally, streamline tractography (using the “tckgen” function in MRtrix3) was performed with parameters initiated at stopping FA value = 0.15, minimal length = 5 mm, and step size = 0.3 mm. Graphical representations of this reconstruction method are detailed elsewhere (Tournier et al., 2012).

**Figure 1:**
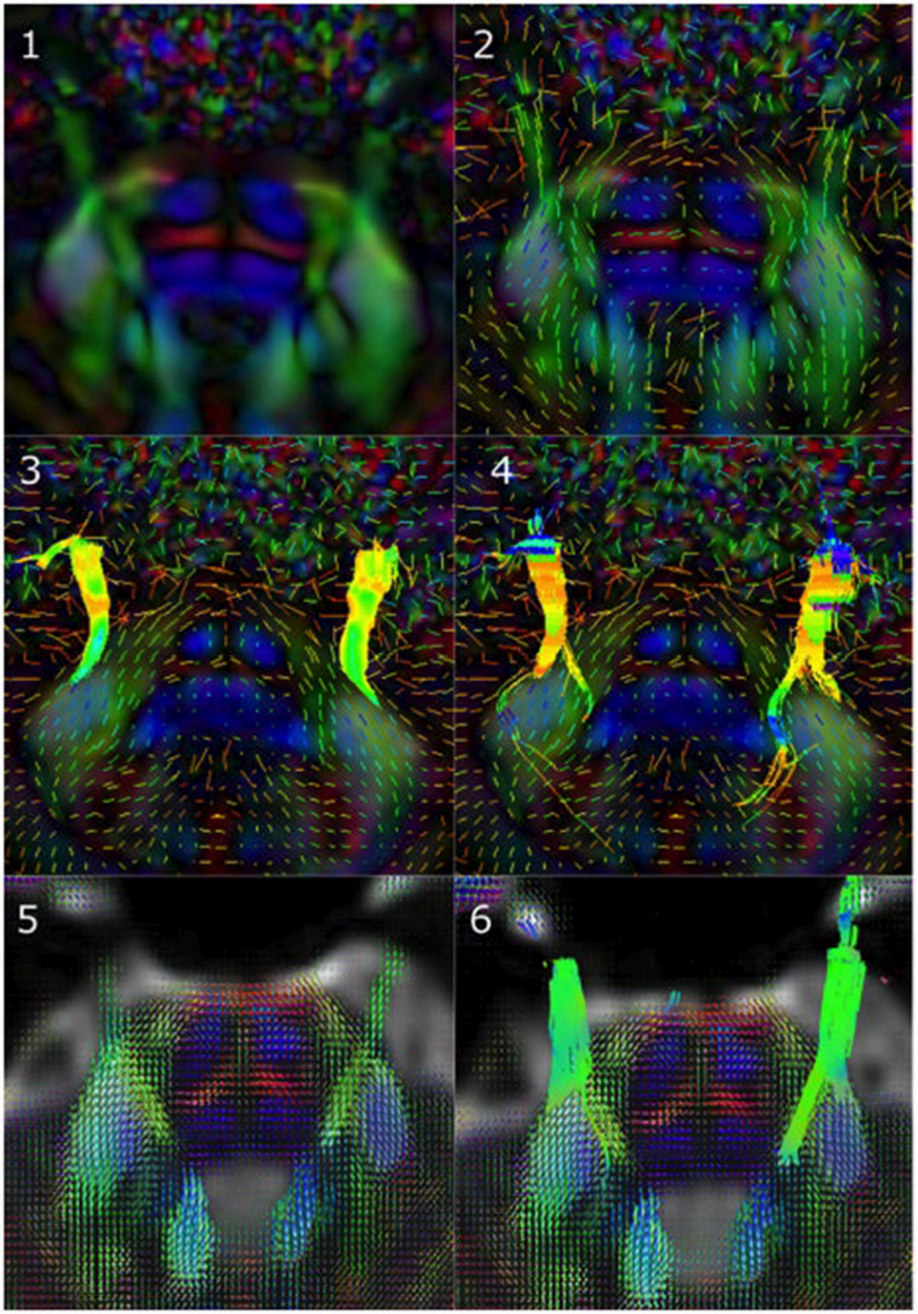
Reconstruction methods differ in how they represent underlying diffusion processes. **(1)** Axial view of a sample tensor map displaying CN V entry into the brainstem, **(2)** Tensors visible as glyphs displaying CN V entry into the brainstem, **(3)** Results from single diffusion tensor tractography (SDT) after placing region-of-interest (ROI) seeds on anterior portions of CN V bilaterally. Generated streamlines do not show their projections to brainstem nuclei, **(4)** Results from EXtended Streamline Tractography (XST) after placing ROI seeds on anterior portions of CN V bilaterally. Streamlines projecting to the area of the nuclei of the trigeminal nerves can be seen, **(5)** Axial view of sample fiber orientation distribution (FOD) map displaying CN V entry into the brainstem, **(6)** Results from streamline tractography on FODs derived from constrained spherical deconvolution (CSD) after placing ROI seeds on anterior portions of CN V bilaterally. Streamlines projecting to the area of the nuclei of the trigeminal nerves can be seen.

Figure 1 illustrates the differences between the three reconstruction methods under investigation in this manuscript. Table 2 details the tracking parameters used for each reconstruction method.

**Table 2:** Tracking parameters used for each reconstruction method.

**Reconstruction method**	**Tracking parameters**
SDT	-stoppingvalue 0.15 -stoppingcurvature 0.8 -minimumlength 5 -clthreshold 0.15 -integrationsteplength 0.1-seedspacing 0.3
XST	-stop aniso:c1,0.1 -stop FA 0.15 -frac 0.1 -radius 0.8 -minlen 5 -step 0.3
CSD	-algorithm SD_STREAM -step 0.3 -angle 45 -rk4 -minlength 5 -cutoff 0.15 -initcutoff 0.15 -force

#### Tractography assessment criteria

The following criteria were used to assess each tractography method:

1. Model creation and tractography time: the duration of time for either tensor-based or FOD-based models to be constructed from preprocessed scans, and the duration of time for subsequent tractography analysis.
2. Anatomical accuracy of tractography output: whether generated WM tracts resembled known anatomical fiber organization.
3. Depiction of CN compression by tumor: whether it was possible to visualize where the tumor in the posterior cranial fossa was compressing the CN of interest.

Table 3 further specifies this assessment criteria.

**Table 3:** Criteria for assessing the results obtained from the three discrete tractography methodologies.

**PROCESSING SPEED**
• Time required for generation of tensor or FOD model
• Time required for tractography once a suitable seed ROI is created
**ANATOMICAL ACCURACY**
**CN V**
• Adequate representation of cisternal segments of CN V
• Adequate representation of projections to brainstem CN V nuclei
• Overall accurate delineation of CN V
**CN VII/VIII**
• Adequate representation of cisternal segments of CN VII/VIII
• Overall accurate delineation of CN VII/VIII
**RELATIONSHIP BETWEEN CN FIBER BUNDLES AND TUMORS IN POSTERIOR CRANIAL FOSSA**
• Adequate visualization of fiber compression in all patients with tumors primarily compressing CN VII/VIII
• Adequate visualization of fiber compression in all patients with tumors primarily compressing CN V

#### Tumor modeling

T1 anatomical images were registered to the DWI using linear registration in 3D Slicer version 4.3 (NA-MIC). The registration procedure was assessed for accuracy in the brainstem area by ensuring that there was accurate alignment of 3 specific anatomical landmarks between DWI and T1 datasets: (a) basilar artery, (b) the ventral bulge of the basis pontis, and (c) the outline of the fourth ventricle.

With clear registration between T1 and DWI datasets achieved, a 3D tumor model was then created in 3D Slicer version 4.3. Briefly, the outline of the tumor was manually traced on each axial slice. This volume was then converted to a 3D model using a Laplacian filter with 30 iterations.

All results from the three reconstruction methods were displayed on either T1 anatomical images or mixed anatomical-diffusion tensor models, which included a 3D model of the posterior cranial fossa tumor. The mixed anatomical-diffusion tensor model represents the tensor map being overlaid on a T1 anatomical image. The results were imported into 3D Slicer for visualization. Importantly, no filtering was applied to the generated tracts. All computations were performed on a Dell Precision T3610 Desktop on a Ubuntu 12.04 LTS OS.

## Results

### Processing time

Imaging datasets including DWI scans from all 10 patients with cerebellopontine angle tumors were successfully corrected for motion-related artifacts. No image datasets were discarded for excessive head motion or associated distortions.

**Figure 2:**
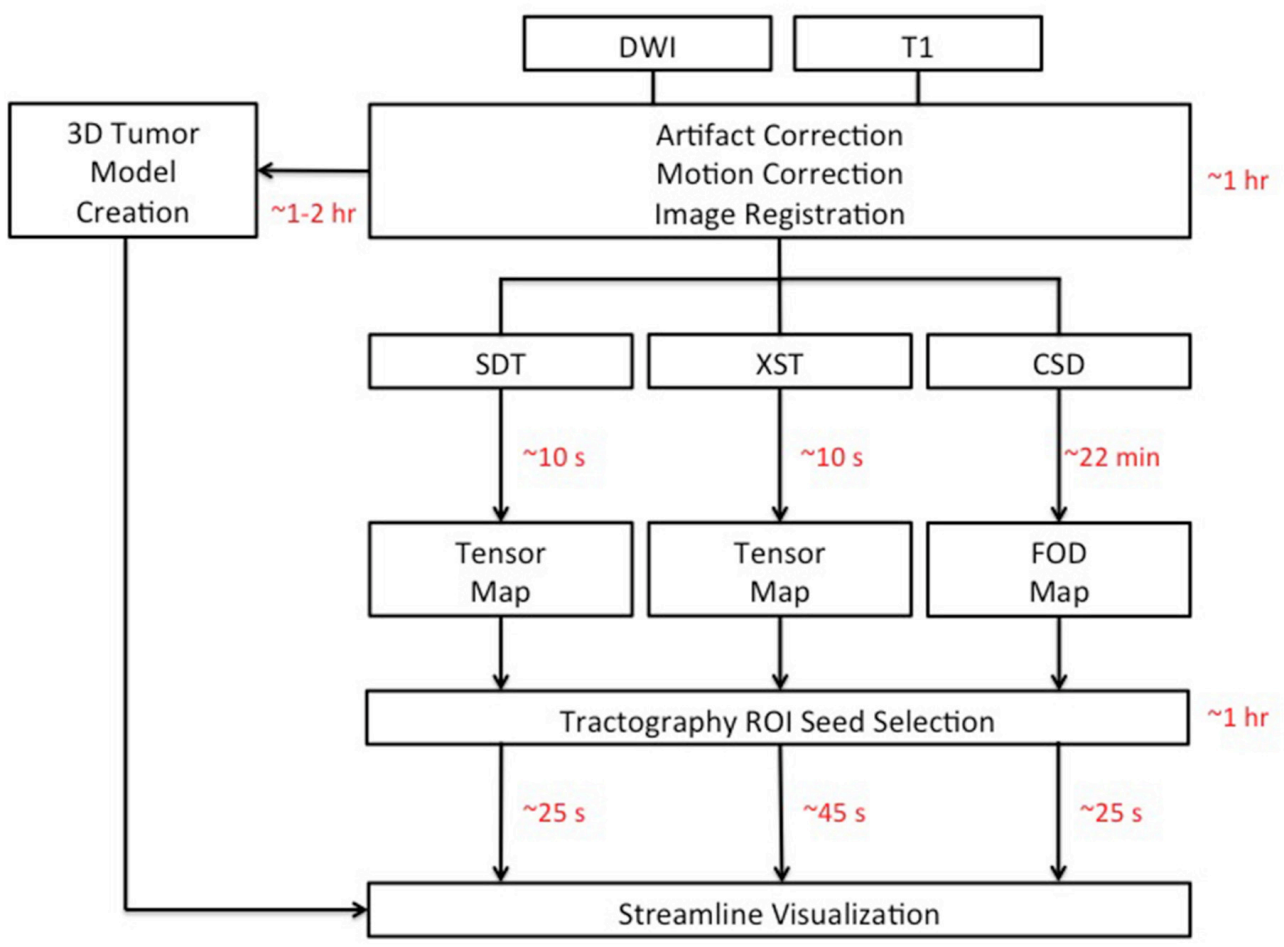
Schematic detailing the analysis pipelines for all three tractography methods: Single Diffusion Tensor Tractography (SDT), EXtended Streamline tractography (XST), Streamline tractography on fiber orientation distributions (FODs) derived from constrained spherical deconvolution (CSD). Acquired diffusion-weighted imaging (DWI) scans were initially corrected for motion- and eddy current-related artifacts. This took ~30 minutes (min) to perform. Registration between T1 anatomical images and DWI images was complete within 1 hour (hr) per patient. 3D models of tumors of the posterior cranial fossa took ~1–2 hr per patient to create. The creation of the tensor map was typically ~10 seconds (s) in duration, where the creation of the FOD map was typically ~22 min in duration. Tractography analysis for cranial nerves (CNs), not directly affected by the presence of a tumor, was complete within 10 min for CN V and 20 min for CN VII/VIII, on average. Tractography analysis for CNs directly affected by the presence of a tumor took longer with 30 min on average for CN V generation in patients with tumors compressing CN V and 1–2 hr in patients with tumors compressing CN VII/VIII. Once suitable seed ROIs had been created, SDT and CSD-based tractography took typically ~25 s on average for CN generation with XST typically taking ~45 s on average. DWI: Diffusion-Weighted Image.

**Figure 3:**
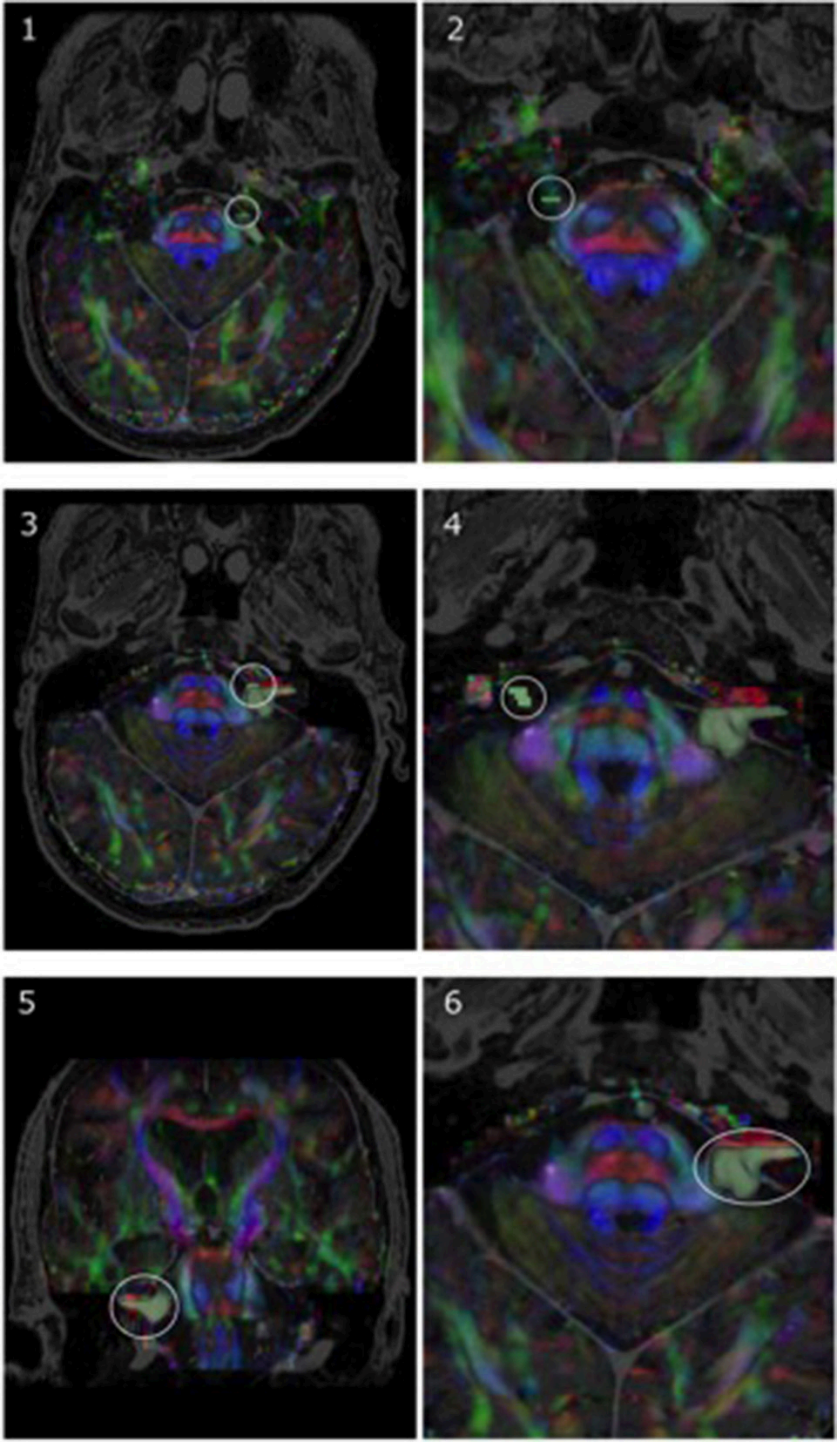
Representative region-of-interest (ROI) seed placement, in green, in one patient with a vestibular schwannoma (P05). **(1, 2)** Highlight ROIs placed on anterior portions of CN V, whereas **(3, 4)** specify the ROI location for CN VII/VIII on both the affected side and side contralateral to the lesion, respectively. Images are displayed on a mixed anatomical-tensor model from a superior view. The mixed anatomical-diffusion tensor model represents the tensor map being overlaid on a T1 anatomical image. Pictures of the vestibular schwannoma from both a coronal perspective **(5)** and zoomed-in axial perspective **(6)** are also displayed.

As processing time is a major concern for neurosurgeons hoping to incorporate DWI-based technology into their practice, we measured processing time for the following steps in this procedure: (i) correcting for motion-related and eddy current-induced artifacts, (ii) Registration between T1 anatomical and diffusion images for anatomical localization, (iii) 3D tumor modeling, (iv), diffusion model (either tensor or FOD) creation, (v) Appropriate seed selection, and (vi) the tracking algorithm itself. These steps, and their associated processing times, are depicted in Figure 2. Representative seeds for tractography are depicted in Figure 3.

Most processing time stemmed from ensuring accurate registration between diffusion and T1 images and accurate tumor model creation [~1 and 1–2 hours (hr) per patient, respectively]. Ensuring appropriate seed selection for CN VII/VIII in patients presenting with vestibular schwannomas (P05–P10) was also time-consuming (1–2 hr per patient for P05–P10). In comparison, suitable seed selection for CN V in patients with tumors primarily compressing this nerve (P01–P04) took ~30 min per patient, indicating that the type of cerebellopontine angle tumor can have a sizeable impact on the time taken for appropriate seed selection. Seed selection for CN V and CN VII/VIII on sides that were not directly affected by the presence of a tumor took ~10 and 20 min, respectively. Correction of motion-related and other image artifacts had an approximate duration of 30 min in total, per patient.

There were also notable processing time differences between the three reconstruction methods. The creation of the tensor map was rapid [typically 10 seconds (s)], compared to the running of the constrained spherical deconvolution resulting in fiber orientation distribution estimates (typically 22 min). For tractography itself, once a suitable seed ROI had been selected, XST took slightly longer for streamline generation (typically ~45 s for CN generation), when compared to both SDT and CSD-based reconstruction methods (typically ~25 s for CN generation from either method).

### Anatomical accuracy

#### CN V

**Figure 4:**
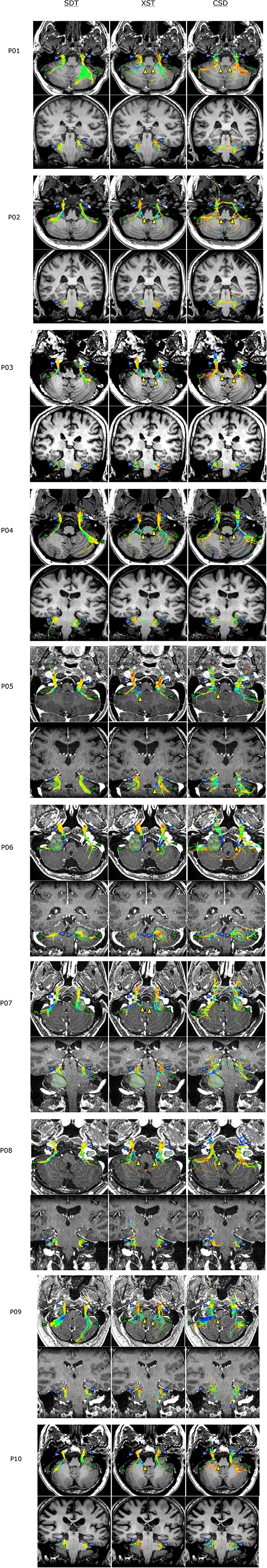
Bilateral CN V generated from each of the three reconstruction methods: Single Diffusion Tensor Tractography (SDT), EXtended Streamline tractography (XST), and streamline tractography on fiber orientation distributions (FODs) derived from constrained spherical deconvolution (CSD) is displayed. Streamlines are displayed overlaid on a T1 anatomical image from a superior and anterior view. Colored triangles indicate particular anatomical landmarks: blue indicates cranial nerves, green indicates superior cerebellar fibers, yellow indicates trigeminal brainstem nuclei. While SDT produced good representations of cisternal segements of CN V (P05–SDT), it was unable to display projections to trigeminal brainstem nuclei (P03–SDT). Both XST and CSD-based tractography could differentiate between superior cerebellar peduncle fibers and trigeminal brainstem nuclei (P03–XST, CSD). SDT also generated aberrant broad projections into the cerebellum when imaging superior cerebellar peduncle fibers (P09–SDT). CSD-based tractography also produced several aberrant fibers when generating superior cerebellar peduncle fibers that encounter trigeminal fibers (P09–CSD).

Figure 4 depicts CN V generated from each of the three reconstruction methods for all 10 patients with tumors of the cerebellopontine angle. All three reconstruction methods accurately delineated portions of CN V, particularly the cisternal and retrogasserian segments. However, there were notable differences between streamlines generated from each method, in terms of accuracy and aberrant streamline generation. While SDT accurately portrayed cisternal portions of CN V, its accuracy diminished closer to the brainstem with its inability to show projections to trigeminal brainstem nuclei and instead solely generated superior cerebellar peduncle streamlines (e.g., Figure 4, P03–SDT). Both XST and CSD-based tractography more accurately demonstrated the distinction between superior cerebellar streamlines and projections to trigeminal brainstem nuclei (Figure 4, P03–XST and CSD). Similarly, SDT sometimes produced inaccurate projections into the cerebellum when tracking superior cerebellar streamlines (Figure 4, P09–SDT). CSD-based reconstruction methods also sometimes produced similar erroneous cerebellar projections (Figure 4, P09 CSDx).

#### CN VII/VIII

**Figure 5:**
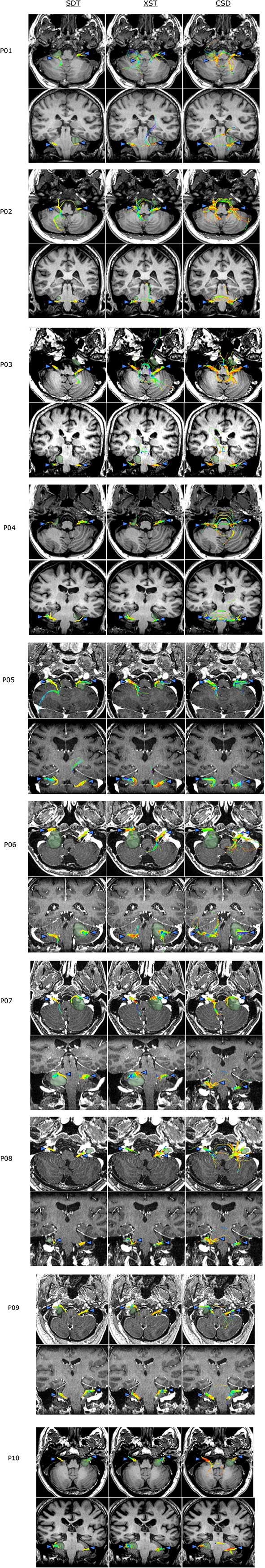
Bilateral CN VII/VIII generated from each of the three reconstruction methods: Single Diffusion Tensor Tractography (SDT), EXtended Streamline tractography (XST), Streamline tractography on fiber orientation distributions (FODs) derived from constrained spherical deconvolution (CSD) is displayed. Streamlines are displayed overlaid on a T1 anatomical image from a superior and anterior view. Colored triangles indicate particular anatomical landmarks: blue indicates cranial nerves, green indicates superior cerebellar fibers, yellow indicates trigeminal brainstem nuclei. SDT was able to display anatomically accurate cisternal portions of CN VII/VIII on sides contralateral to lesions (P06–SDT). However, XST and CSD-based tractography produced longer sections of CN VII/VIII (P10–SDT, XST, CSD). On affected sides, SDT could only generate small cisternal segments (P08–SDT). XST and CSD-based tractography depicted longer cisternal segments (P08–XST, CSD). However, CSD-based tractography also generated several anatomically inaccurate fibers when generating CN VII/VIII in this patient (P08–CSD).

Figure 5 depicts CN VII/VIII generated from each of the three reconstruction methods. Similarly to CN V, all three methods successfully portions of CN VII/VIII, particularly the cisternal segment, with XST and CSD-based reconstruction methods tending to generate longer portions of the CNs, particularly CNs VII/VIII that were not in the proximity of a tumor, when compared to SDT (Figure 5, P02, P03, P04, P06, P10).

**Figure 6:**
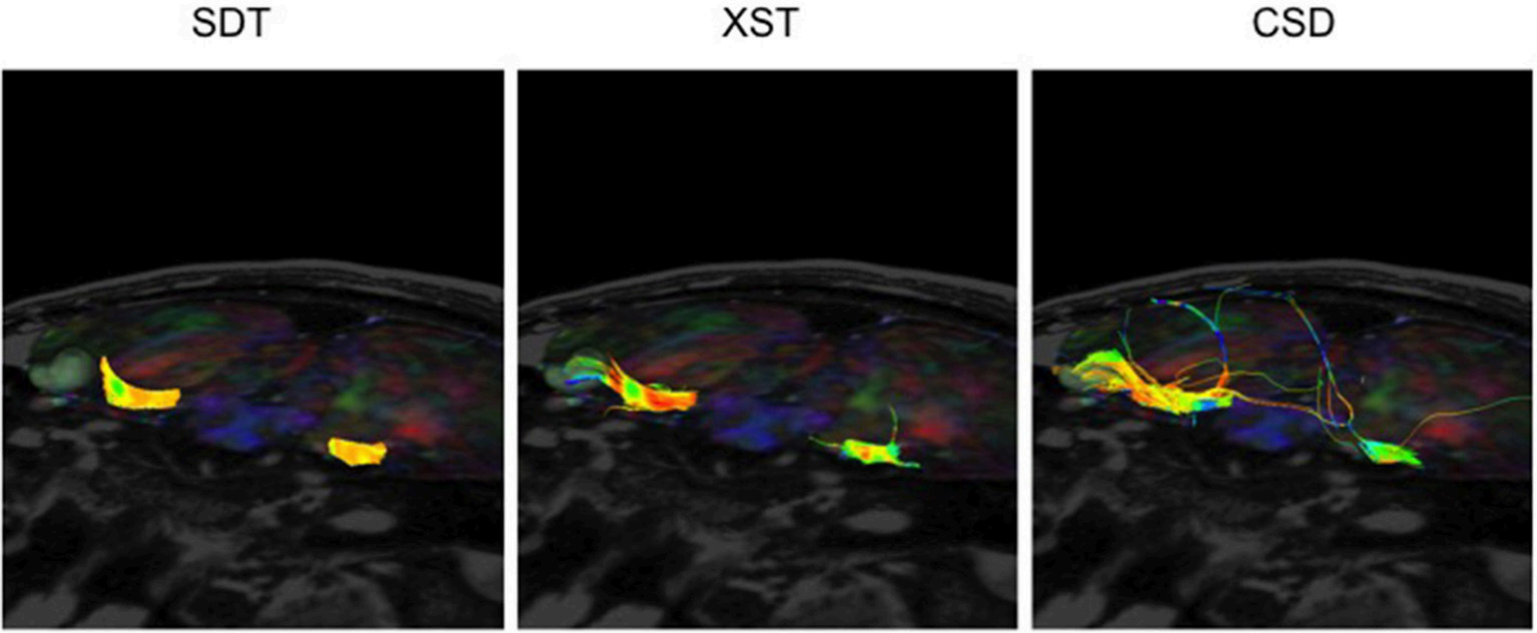
CN VII/VIII generation from all three reconstruction methods in the same patient–P08. SDT could not show CN VII/VIII curving around tumor to anterior surface. However, CSD-based tractography produced more spurious fibers. Single Diffusion Tensor Tractography (SDT), EXtended Streamline tractography (XST), Streamline tractography on fiber orientation distributions (FODs) derived from constrained spherical deconvolution (CSD). Fibers are displayed from a superior-anterior perspective toward the brainstem. Images are displayed on a mixed anatomical-tensor model. The mixed anatomical-diffusion tensor model represents the tensor map being overlaid on a T1 anatomical image.

On the other hand, while CSD-based reconstruction methods were able to provide detailed reconstructions of the CNs, it sometimes generated a notable amount of spurious streamlines, when compared to the output from either other method. The depictions of CN VII/VIII in P08 illustrate this point (Figure 6). CSD-based reconstruction methods produced several aberrant streamlines that emerged superiorly, anteriorly and posteriorly from the generated cisternal segment of CN VII/VIII, between its exit from the brainstem and where it came into contact with tumor, compared to the two other methods.

### Depiction of tumor/CN relationship

#### CN V

All three methods satisfactorily illustrated this relationship in the four patients presenting with tumors primarily affecting CN V (Figure 4, P01, P02, P03, P04). It was possible to clearly see where the generated CN V came into contact with the generated 3D tumor model and would have allowed for clear tumor resection borders to be defined.

#### CN VII/VIII

**Figure 7:**
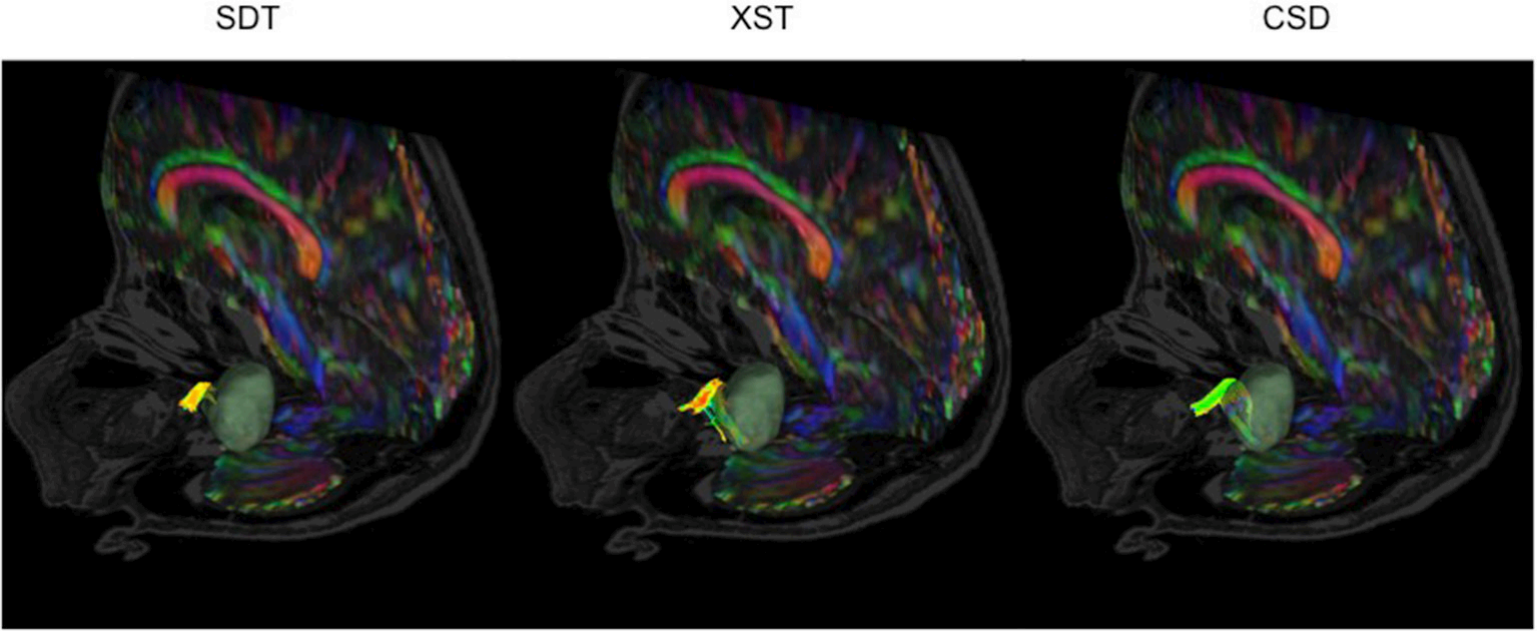
CN VII/VIII generation from all three reconstruction methods in a patient with a vestibular schwannoma compressing CN VII/VIII-P06. SDT was only able to show portions of CN VII/VIII anterior to the tumor, where both XST and CSD-based tractography could show CN VII/VIII curving under the vestibular schwannoma. Single Diffusion Tensor Tractography (SDT), EXtended Streamline tractography (XST), Streamline tractography on fiber orientation distributions (FODs) derived from constrained spherical deconvolution (CSD). Fibers are displayed from a posterior-lateral perspective toward the brainstem. Images are displayed on a mixed anatomical-tensor model. The mixed anatomical-diffusion tensor model represents the tensor map being overlaid on a T1 anatomical image.

Similarly, it was possible to visualize where the lesion was compressing CN VII/VIII, using all three methods under consideration, in most of the patients presenting with vestibular schwannomas (Figure 5, P05, P07, P08, P09, P10). Notably, in one patient (Figure 7, P06), only XST and CSD-based reconstruction methods could depict CN VII/VIII curving under the posterior cranial fossa tumor. SDT was not able to do so and simply displayed a portion of CN VII/VIII anterior to the tumor and not curving underneath it.

Table 4 illustrates results, for each tractography method, in relation to the tractography assessment criteria.

**Table 4:** Summary of the results from the three tractography methodologies under consideration.

**Methods**	**SDT**	**XST**	**CSD**
**PROCESSING SPEED**			
Time required for generation of tensor or FOD model	10 sec	10 sec	22 min
Time required for tractography once a suitable seed ROI is created	25 sec	45 sec	25 sec
**ANATOMICAL ACCURACY**			
**CN V**			
Adequate representation of cisternal segments of CN V	Yes	Yes	Yes
Adequate representation of projections to brainstem CN V nuclei	No	Yes	Yes
Overall accurate delineation of CN V	No	Yes	Yes
**CN VII/VIII**			
Adequate representation of cisternal segments of CN VII/VIII	Yes	Yes	Yes
Overall accurate delineation of CN VII/VIII	No	Yes	Yes
**RELATIONSHIP BETWEEN CN FIBER BUNDLES AND TUMORS IN POSTERIOR CRANIAL FOSSA**			
Adequate visualization of fiber compression in all patients with tumors primarily compressing CN VII/VIII	No	Yes	Yes
Adequate visualization of fiber compression in all patients with tumors primarily compressing CN V	Yes	Yes	Yes

*ROI, region-of-interest; Single Diffusion Tensor Tractography (SDT), EXtended Streamline Tractography (XST), Streamline tractography on fiber orientation distributions (FODs) derived from constrained spherical deconvolution (CSD)*.

## Discussion

XST appears to be the optimal reconstruction method for CN generation in a neurosurgical context, due to its speed and accuracy, when compared to the other methods under consideration here. XST uses the conventional tensor map and is nearly as rapid as the other techniques under investigation here in terms of tracking time. XST was able to generate larger anatomically accurate portions of the CN of interest, compared to SDT—the most commonly used reconstruction method for neurosurgical purposes. Further to this, it also could differentiate representations of fiber tracts, while not generating as many aberrant streamlines, as CSD-based streamline tractography did.

Here, we found that while SDT was the most accessible and easy-to-use program (i.e., tracking can be performed through a Graphical User interface) of all three reconstruction methods under review, its results oftentimes only displayed smaller portions of the CNs of interest, compared to the two other methods under consideration and could not distinguish between representations of CN and cerebellar peduncle fibers. Such inaccurate results from SDT reconstruction methods may pose a major problem for neurosurgeons who would need to know exactly where CN V exits the brainstem and the relationship between the CN itself and nearby cerebellar peduncular fibers. Also, in one patient with a vestibular schwannoma, SDT reconstruction methods could not adequately depict the relationship between CN VII/VIII and tumor, where the other methods were able to do so. This ability to depict CN VII/VIII compression may be related to how both XST and CSD-based methods can adequately account for crossing fiber populations, where SDT cannot. Thus, this complex fiber arrangement of CN VII/VIII emerging from under the posterior cranial fossa tumor and projecting laterally to the cochlear region is only visualized from employing more advanced reconstruction methods. SDT, with its inherent assumption of one fiber bundle per voxel, could not adequately image the course of this fiber bundle as it curved under the tumor and should be a cautionary note for neurosurgeons implementing SDT through available software programs in their clinical practice.

Such complex fiber arrangements appear to be problematic for SDT to resolve, with its assumption of one fiber orientation per voxel. Previous work has illustrated such difficulties in patients with tumors in other parts of the brain. Kuhnt et al. (2013a) reported that SDT could not adequately resolve fibers in the vicinity of gliomas occurring in language-associated cortical areas. Similarly, Kuhnt et al. (2013b) observed that optic radiation fibers were not accurately depicted when SDT was applied to diffusion MRI data derived from patients with gliomas in the temporal lobe. Chen et al. (2015) demonstrated that disruptions to the arcuate fasciculus were not adequately illustrated using SDT in patients with tumors with peritumoral edema in language-related areas. Anatomically inaccurate depictions of corticospinal tracts derived from SDT in patients with tumors in the vicinity of the motor cortex have also been well described (Qazi et al., 2009; Farquharson et al., 2013; Chen et al., 2016).

Interestingly, while these limitations of the tensor model have been acknowledged for some time and how they may result in false tracking results, most software programs offering tractography tend to still mostly depend solely on it (Soares et al., 2013). This is since alternative HARDI algorithms, which can accommodate crossing fiber populations, require longer DWI scans with a large number of directions, which may be difficult to routinely implement in a clinical environment. Further to this, the application of advanced methods comes with its own set of challenges including the correction of distortion effects (Nimsky, 2014; Nimsky et al., 2016). The DWI scans used in this study, which allowed for implementation of HARDI algorithms, did not require a relatively large amount of time for acquisition (~17.5 min in duration) and were collected as part of standard clinical protocol. This may be attractive to other neurosurgical groups interested in implementing HARDI-based tractography approaches.

Nonetheless a common consensus is still lacking as to which HARDI algorithm offers the best trade-off between scan acquisition time and tractography output for a clinical population. It appears that streamline tractography involving FODs derived from a CSD approach are demonstrating superior results compared to other HARDI-based models, such as q-ball imaging and “ball and stick” models, for larger WM pathways at least (Wilkins et al., 2015). However, we found that when it comes to smaller WM tracts, a multi-tensor reconstruction method, XST, appears to produce more reliable tracts from a conventional tensor approach—an approach that is widely available to neurosurgeons through currently accessible DWI software programs and takes a small amount of time (typically 10 s in this study) to compute. This reliability may stem how its propagation phase operates—the tensor, whose principal eigenvector has the least deviation from the incoming trajectory, is chosen for the next step during reconstruction. This approach helps ensure there is a consistent streamline trajectory, particularly when intravoxel multiple fibers are encountered.

It was interesting to note that while both XST and CSD-based reconstruction methods used different approaches, tensor- and FOD-based ones, respectively, they shared similar outputs. Both were able to differentiate between CN V and cerebellar tracts in a number of patients and clearly define where CN V exited the brainstem. In all cases, both methods were able to demonstrate the relationship between the posterior cranial fossa and affected CN. However, XST produced far fewer spurious streamlines than CSD-based streamline tractography leading to our recommendation that it should be the HARDI algorithm of choice for neurosurgical groups aiming to generate CNs in patients with posterior cranial fossa tumors. Previous demonstrations of CSD-based tractography have tended to involve probabilistic-based algorithms (Farquharson et al., 2013; Palesi et al., 2015). Here, we opted to use the CSD-based deterministic tractography, due to its superior processing speed for neurosurgical purposes (Qazi et al., 2009), which may explain the occurrence of these spurious fibers. While we acknowledge that it is possible to limit the number of tracks generated from CSD-based streamline tractography, as well as creating inclusion/exclusion ROIs, this raises other issues about choosing appropriate thresholds and criteria, all of which can take away from optimal tracking in a clinical setting, where rapid processing and interpretation are essential. Here, XST provided clear depictions of CNs without a reliance on the inclusion of extra criteria, in addition to those specified in the initial tractography command, making it an attractive option for neurosurgeons hoping to implement HARDI-based approaches in a time-dependent manner. As such reconstruction methods continue to be integrated in neurosurgical settings (Sammartino et al., 2016; Essayed et al., 2017), the selection of appropriate methods for accurate visualization of nerve fibers will continue to be of paramount importance.

## Conclusions

These results suggest that a HARDI-based reconstruction method, XST, is currently the optimal option for visualizing how CNs V and VII/VIII are affected by the development of a tumor in the posterior cranial fossa. While the other reconstruction methods provide reasonable results, XST allows for a rapid tracking procedure where crossing fiber populations can be adequately represented while minimizing the number of anatomically inaccurate fiber representations generated. As XST works with the conventional tensor-based map, neurosurgical teams can easily incorporate this approach into their current analysis pipeline for reliable tract generation in a clinical environment. We would recommend that clinicians continue with their clinically approved SDT analysis but supplement it with these more advanced reconstruction methods for more accurate CN visualization.

## Ethics statement

This study was carried out in accordance with the recommendations of University Health Network (UHN) Research Ethics Board with written informed consent from all subjects. All subjects gave written informed consent in accordance with the Declaration of Helsinki. The protocol was approved by the University Health Network (UHN) Research Ethics Board.

## Author contribution

BB and MH: Assisted with the design and conception of this work. BB, DD, and MH: Assisted with the acquisition of data for this work. BB, DC, and MH: Assisted with the data analysis for this work. BB, FS, EW, and MH: Assisted with the interpretation of data for this work. All authors were involved in the drafting of this work and giving final approval before submission. All authors agree to be accountable for all aspects of this work.

### Conflict of interest statement

The authors declare that the research was conducted in the absence of any commercial or financial relationships that could be construed as a potential conflict of interest.
